# TRPV1 and TRPA1 Mediate Peripheral Nitric Oxide-Induced Nociception in Mice

**DOI:** 10.1371/journal.pone.0007596

**Published:** 2009-10-29

**Authors:** Takashi Miyamoto, Adrienne E. Dubin, Matt J. Petrus, Ardem Patapoutian

**Affiliations:** 1 Department of Cell Biology, The Scripps Research Institute, La Jolla, California, United States of America; 2 Department of Neurobiology, Genomics Institute of the Novartis Research Foundation, San Diego, California, United States of America; SIU School of Medicine, United States of America

## Abstract

Nitric oxide (NO) can induce acute pain in humans and plays an important role in pain sensitization caused by inflammation and injury in animal models. There is evidence that NO acts both in the central nervous system via a cyclic GMP pathway and in the periphery on sensory neurons through unknown mechanisms. It has recently been suggested that TRPV1 and TRPA1, two polymodal ion channels that sense noxious stimuli impinging on peripheral nociceptors, are activated by NO in heterologous systems. Here, we investigate the relevance of this activation. We demonstrate that NO donors directly activate TRPV1 and TRPA1 in isolated inside-out patch recordings. Cultured primary sensory neurons display both TRPV1- and TRPA1-dependent responses to NO donors. BH4, an essential co-factor for NO production, causes activation of a subset of DRG neurons as assayed by calcium imaging, and this activation is at least partly dependent on nitric oxide synthase activity. We show that BH4-induced calcium influx is ablated in DRG neurons from TRPA1/TRPV1 double knockout mice, suggesting that production of endogenous levels of NO can activate these ion channels. In behavioral assays, peripheral NO-induced nociception is compromised when TRPV1 and TRPA1 are both ablated. These results provide genetic evidence that the peripheral nociceptive action of NO is mediated by both TRPV1 and TRPA1.

## Introduction

Nitric oxide (NO) is a gaseous signaling molecule generated from arginine and oxygen by nitric oxide synthases (NOS) [1]. NO plays essential roles in various biological processes including vascular signaling, immune responses, neurotransmission, and pain sensation/sensitization [2]–[3]. NO signaling is carried out by at least two separate pathways. In the first pathway, NO stimulates soluble guanylyl cyclase (sGC) to increase cyclic guanosine monophosphate (cGMP), which in turn modulates a variety of downstream signaling targets [4]. In the second pathway, NO covalently and reversibly forms adducts with free thiols of cysteine residues within proteins and hence directly modifies protein function [5]. Although functional significance of this S-nitrosylation has not been as well established as the NO-cGMP pathway, accumulating evidence suggests that it is a widespread mechanism of NO action [5]–[6].

NO plays essential roles in development and maintenance of pain in response to inflammation and injury through its actions at both peripheral and central sites [3], [7]. NOS enzymes are expressed in peripheral sensory dorsal root ganglia (DRG) and central spinal cord neurons, and are upregulated during inflammation and injury [8]–[9]. The importance of NO production in inflammatory and neuropathic pain has been well documented through pharmacologic and genetic approaches [10]–[11]. Sensitization of central spinal cord neurons by NO contributes to hyperalgesia (increased sensitivity to painful stimuli) caused by inflammation and injury. For example, intrathecal administration of L-arginine (a substrate of NOS) induces hyperalgesia [12], while intrathecal administration of NOS blockers or NO scavengers block NMDA-induced hyperalgesia and neuropathic pain [12]–[13]. The mechanism of NO action on central spinal cord neurons in pain transmission is mainly dependent on the NO-cGMP signaling pathway [3].

In addition to modulating central neuronal activity, NO contributes to acute and chronic pain at the periphery [7]. For example, intracutaneous perfusion of NO causes pain sensation in humans [14]. Local NOS inhibition blocks chronic constriction injury (CCI)-induced neuropathic pain [15], prostaglandin E_2_-induced hyperalgesia [16] and paw edema caused by bradykinin or Substance P injection [17]–[18]. Furthermore, GTP cyclohydrolase, the rate-limiting enzyme for BH4 (tetrahydrobiopterin) synthesis, has been shown to regulate pain sensitivity and persistence in the periphery via BH4, an essential cofactor for NO production by NOS [19]. Remarkably, little is known about the molecular mechanism of NO's involvement in peripheral pain.

It has recently been suggested in heterologous systems that several members of the transient receptor potential (TRP) channels, including two peripherally-expressed polymodal nocisensors TRPV1 and TRPA1, are activated by NO donors [20]–[22]. However, thermoTRPs have proven to be promiscuous receptors, and determining a physiological relevance of TRP channel activation requires genetic or pharmacological evidence. Here, we investigated if the activation of the TRP ion channels by NO could underlie NO's involvement in peripheral pain, mainly focusing on primary sensory neurons and behavioral consequences of NO action.

## Results

### Nitric Oxide Donor Activates Primary DRG Neurons

**Figure 1:**
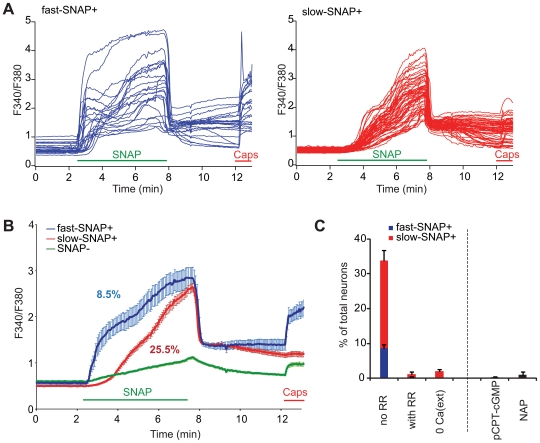
NO donor activates primary cultured DRG neurons. (A, B) Responses of primary cultured DRG neurons assessed by ratiometric calcium imaging. Fluorescent ratios (F340/F380) of individual neurons (A) and averaged traces (B) are shown. Fast-SNAP+ neurons display significantly increased intracellular calcium upon NO donor application within one minute (blue traces), and slow-SNAP+ neurons after one minute (red traces). Neurons without significant increases in intracellular calcium are also shown (B, green trace). Proportions of fast- and slow-SNAP+ in (B) are shown as percentages. DRG neurons were challenged with SNAP (3 mM) for 5 min (green bar). Capsaicin (Caps, 10 µM, red bar) was applied at the end as a control. (C) Summary of responses of primary cultured DRG neurons assessed by ratiometric calcium imaging. Proportions of neurons activated by SNAP (three left columns; subdivided into fast-SNAP+ (blue) and slow-SNAP+ (red) responders), and pCPT-cGMP (2 mM) and NAP (3 mM; non-NO releasing isomer of SNAP) (two right black columns) are shown. SNAP induced intracellular calcium responses were sensitive to Ruthenium red (“with RR”) and required extracellular calcium (“0 Ca(ext)”). Cells were exposed to RR (10 µM) and calcium depleted saline 3 min prior to and throughout SNAP application and washed out prior to capsaicin addition. The proportion responsive to capsaicin was normal in these experiments.

NO, presumably through direct activation of nociceptors, causes acute pain in humans [14]. We thus sought to determine if NO activates cultured DRG neurons using compounds that spontaneously release NO. To assay large numbers of neurons simultaneously, we used ratiometric calcium imaging which detects global changes in cytoplasmic calcium levels and assesses both direct (e.g., modulation of a putative calcium-permeable NO receptor) and indirect (e.g., release of calcium from intracellular stores and/or downstream activation of calcium-permeable ion channels) processes. Interestingly, we observed intracellular calcium increase in approximately ∼30% of total neurons upon application of the NO donor SNAP (S-nitroso-N-acetylpenicillamine; 3 mM) (Fig. 1A, B and Table 1). Lower concentrations of SNAP (≤1 mM) did not significantly alter calcium levels in DRG neurons [23]. A congener of SNAP lacking the releasable NO group (NAP: N-acetylpenicillamine; 3 mM) did not activate DRG neurons, consistent with NO-dependent activation (Fig. 1C). SNAP had little effect in the absence of extracellular calcium ions (Fig. 1C), suggesting that the signal was largely due to influx of calcium through calcium-permeable ion channels. Based on the kinetics of SNAP-evoked activation, we defined two apparently distinct populations of neurons activated by SNAP by setting arbitrary thresholds: those revealing a significant increase in intracellular calcium within one minute after SNAP application (“fast-SNAP+” neurons, Fig. 1A left, 1B blue), and those exhibiting a significant increase after one minute (“slow-SNAP+” neurons, Fig. 1A right, 1B red). Soluble guanylyl cyclase (sGC), a well-characterized molecular target of NO which causes an increase in intracellular cGMP in many cell types [4], is not expressed in DRG neurons [24]. We confirmed that NO donor activates DRG neurons in a cGMP-independent manner using the membrane-permeable cGMP analogue pCPT-cGMP (8-(4-Chlorophenylthio)-guanosine 3′,5′-cyclic monophosphate) [25] (Fig. 1C). We next tested whether TRP channels might mediate the NO donor-induced calcium response in DRG neurons by determining the sensitivity of the response to the non-selective TRP channel antagonist, ruthenium red (RR). RR almost completely blocked the NO donor-induced increase in calcium levels when applied before application of SNAP (Fig. 1C, and Table 1). These results suggest that NO released from SNAP activates a subset of DRG neurons through RR-sensitive ion channel(s).

**Table 1:** NO donor activates DRG neurons via TRPV1 and TRPA1.

%	SNAP	SNAP fast	SNAP slow	Capsaicin	Mustard Oil
**Wildtype**	33.9±2.9	8.7±1.0	25.2±2.9	31.5±0.6	
**Wildtype+RR** *	1.2±0.8	0.2±0.2	1.0±0.6	12.9±3.3	
**Wildtype+BCTC** *	19.2±1.5	1.7±0.9	17.5±2.3		37.0±2.0
**Wildtype+AP-18** *	11.9±1.5	10.3±1.6	1.5±0.5	18.7±2.6	
**TRPV1^−/−^**	0.9±0.3	0.0±0.0	0.9±0.3	0.0±0.0	26.6±3.0
**TRPA1^−/−^**	7.9±1.0	6.9±1.0	1.0±0.2	35.0±2.2	0.2±0.2
**V1^−/−^ A1^−/−^** **	1.3±0.1	0.0±0.0	1.3±0.1	0.0±0.0	0.0±0.0

Summary of the percent of responsive cultured DRG neurons to SNAP (3 mM), capsaicin (10 µM), and mustard oil (0.5 mM) from wildtype (with or without antagonists), TRPV1^−/−^, TRPA1^−/−^, and TRPV1^−/−^TRPA1^−/−^ (V1^−/−^ A1^−/−^) animals.

* Ruthenium Red (10 µM), BCTC (1 µM), or AP-18 (100 µM) were applied 3 min prior to SNAP application until SNAP washout.

** ATP (100 µM) was applied in the last of the experiment as a positive control. Approximately 20% of total DRG neurons were activated by application of ATP.

### TRPV1 and TRPA1 Are Directly Activated by NO Donors

We next sought to identify the molecular target(s) of NO donors within DRG neurons. TRPV1 and/or TRPA1 are prime candidate receptors, as they are expressed in nociceptors, are blocked by RR, and are known to be sensitive to reactive chemicals via covalent modification of cysteine residues [26]–[28]. Arguing against this, Hinman et al. stated that NO does not activate TRPA1 [26]. However, activation of recombinant TRPA1 and TRPV1 by NO donors in calcium imaging experiments has recently been reported [20]–[22]. Therefore, we re-examined if recombinant TRPV1 and/or TRPA1 could serve as molecular sensors for NO.

**Figure 2:**
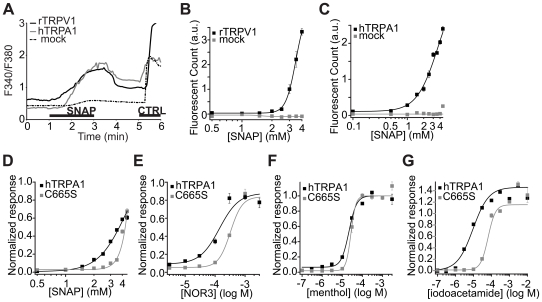
NO donors induce calcium influx in cells expressing TRPV1 or TRPA1. (A) SNAP (2 mM) increases intracellular calcium (F340/F380) in rTRPV1- and hTRPA1-expressing CHO cells (black and grey lines, respectively) but not YFP-positive mock transfected CHO cells (dashed line). Capsaicin (1 µM), mustard oil (250 µM), or ATP (100 µM) was applied as a control (“CTRL”) for TRPV1-, TRPA1-expressing, or mock-transfected cells, respectively. Averages of at least 30 cells are shown. (B, C) Concentration-dependence of SNAP responses in rTRPV1- (B) and hTRPA1- (C) expressing HEK293T cells obtained by FLIPR. a.u.: arbitral unit. (D–G) Normalized concentration response curves to SNAP (D), NOR3 (E), menthol (F), and iodoacetamide (G) of HEK293T cells transiently expressing hTRPA1 (black) and C665S hTRPA1 (grey) determined by FLIPR.

SNAP at 2 mM increased intracellular calcium in Chinese Hamster Ovary (CHO) cells expressing TRPV1 or TRPA1 (Fig. 2A). SNAP produced a concentration-dependent activation of TRPV1 and TRPA1 in Human Embryonic Kidney (HEK293T) cells in experiments using the fluorometric imaging plate reader (FLIPR) (Fig. 2B, C). Due to the presence of a large background response to >5 mM SNAP, we were unable to obtain full concentration-response curves (but see response curve for NOR3: (±)-(E)-Ethyl-2-[(E)-hydroxyimino]-5-nitro-3-hexeneamide, another NO donor, below, Fig. 2E). Activation of heterologously-expressed TRPV1 and TRPA1 by SNAP (up to 4 mM) was completely blocked by RR [23].

NO is known to reversibly form covalent adducts to free thiol groups of cysteine residues via S-nitrosylation [1]. TRPA1 and TRPV1 are both activated by electrophilic molecules through covalent modification of cysteine residues in the N-terminus [26]–[28]. We next investigated whether cysteine residues of TRPA1 are required for NO donor-dependent activation. Three cysteines and a lysine in the N-terminal region have been implicated in responses to reactive chemicals [26]–[27]. We therefore tested whether these residues are required for NO activation of TRPA1. As expected, both the triple cysteine (“3C”: C621S, C641S, C665S) and the 3C with single lysine (“3CK”and additional K710R) TRPA1 mutants revealed decreased sensitivity to the cysteine alkylating reagent iodoacetamide (IA) as well as SNAP [23]. However, we found the sensitivity of these clones to the non-reactive agonists menthol [29] and 2-APB (2-Aminoethoxydiphenyl borate) to be moderately (3C) or severely (3CK) compromised [23]. In an attempt to identify TRPA1 mutants that have specific deficits in sensitivity to reactive chemicals (NO donors and/or IA) but not non-reactive agonists (i.e., with little/no general defects in overall channel function), we generated the three described individual cysteine mutants of TRPA1. Compared to wildtype TRPA1, C665S showed markedly right-shifted responses to two chemically distinct NO donors (SNAP and NOR3) and IA, while displaying relatively normal responses to menthol (Fig. 2D–G). This suggests that NO donor-evoked responses are at least partly modulated through cysteine 665, although clearly other residues/mechanisms are also involved. C621S showed relatively normal responses to SNAP, while C641S showed slightly decreased sensitivity to both NO donor and menthol, and thus is difficult to interpret [23]. Since histidines and cysteines are prime targets of NO [30], we also tested TRPA1 mutants with every histidine and cysteine individually mutated [31]. However, we found no additional residues specifically required for responses to NO but not for non-reactive agonists [23] (see Discussion).

While TRPA1 appears to be indiscriminately activated by most reactive chemicals, TRPV1 is less promiscuous [26]–[28], [32]. For TRPV1, C157 is reported to be required for allicin activation; and C616 and C621 (in the putative pore region) are proposed to be required for NO donor activation [20], [28], (however, also see [33]). We found that NO was still able to activate TRPV1-C157. Furthermore, the TRPV1 mutants;C616W:C621S (double cysteine mutant), C616A, and C621A each revealed normal NO donor-induced responses when normalized to capsaicin responses [23], contrary to the findings of Yoshida and colleagues (see Discussion) [20]. We also tested TRPV1 mutants with mutated histidine residues [34], but none of them showed specific deficits to SNAP [23].

**Figure 3:**
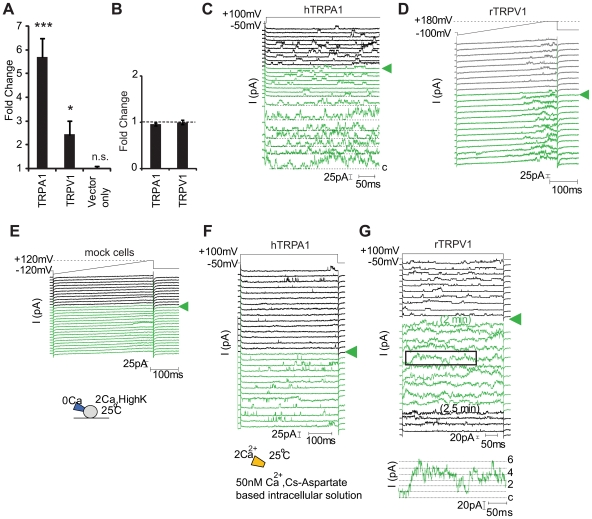
NO donor directly activates TRPV1 and TRPA1. SNAP (3 mM) increases TRP channel activity in cell-attached (A–E) and excised inside out patches (F, G). (A, B) The average fold change induced by SNAP (3 mM) (A) or by the inactive congener NAP (3 mM) (B) in cell-attached patches expressing TRPA1 (n = 11 for (A), n = 4 for (B)), rTRPV1 (n = 4 for (A), n = 3 for (B)) and endogenous channels from YFP-positive vector transfected cells (“Vector only”; n = 3) is shown. Quantification of SNAP induced currents involved taking the difference between the averaged current obtained at a single voltage for all traces before and during SNAP application and the basal current level determined by averaging blank sweeps containing no TRP activity) during these same periods. The fold increase in current induced by SNAP is the leak subtracted SNAP-induced current normalized to the leak subtracted control current prior to SNAP addition (see Materials and Methods for more detail). (C) A cell-attached patch containing at least five hTRPA1 channels was challenged with a step depolarization from the resting membrane potential (near 0 mV) (V_pipette_ from +50 to −100 mV). Cell was exposed to SNAP (3 mM) beginning at the arrow (green traces);latency was 40 sec. (D) A cell-attached patch containing at least three rTRPV1 channels was challenged with a voltage ramp from −120 to +180 mV (V_pipette_ from +120 to −180 mV) over 300 msec to visualize voltage-gated channel activity. Cell was exposed to NAP (3 mM, grey sweeps) initially and then SNAP (3 mM, green) at the time indicated by the green arrow. (E) A YFP-positive HEK293T cell expressing vector was challenged. No TRP-like SNAP-induced channel activity was observed over 2 min. Cartoon of experimental protocol is shown. (F, G) SNAP (3 mM) activated hTRPA1 (F) and rTRPV1 (G) in excised inside out patches. Cartoon of experimental protocol is shown. The inset in (G) shows currents in the boxed area at higher gain to indicate that the activity is consistent with the gating of at least 6 single channels. n.s. not significant.

To verify our calcium imaging results, and to address whether NO-activation is membrane delimited, we next used electrophysiological methods to directly determine whether SNAP could activate recombinant TRPA1 and TRPV1. Initially we monitored TRPV1 and TRPA1 channel activity in the cell-attached configuration which minimally disrupts the intracellular milieu of the cell in the absence and presence of extracellularly applied 2.5–3 mM SNAP. In accordance with calcium imaging and FLIPR experiments, TRPA1 channel activity was significantly enhanced 5.7-fold (p<0.0001; One sample *t*-test) in 84% of patches during exposure to SNAP (Fig. 3A, C). SNAP also increased TRPV1 activity in this configuration by 2.5-fold (p<0.05) in 67% of patches (Fig. 3A, D). Vector transfected cells revealed no detectable change in outward current (i.e., fold change  = 1) (Fig. 3A, E). Prolonged exposure to the non-NO releasing congener of SNAP (NAP; 3 mM) elicited no change in TRPA1 and TRPV1 channel activity for at least twice the average latency to observe SNAP effects (Fig. 3B; p>0.2 for TRPA1 and p>0.7 for TRPV1). To determine whether cytoplasmic constituents were required for SNAP-dependent activation, we recorded from TRPA1 and TRPV1 in excised inside-out patches in the absence of GTP (guanosine-5′-triphosphate). Robust activation of both TRPA1 (n = 3; 8.5+/−2.7 fold) and TRPV1 (n = 2; 3.6 and 7.2 fold) were clearly observed in this configuration (Fig. 3F, G). These results support the hypothesis that TRPV1 and TRPA1 are directly activated by NO.

### NO Donor Activates DRG Neurons via TRPV1 and TRPA1

**Figure 4:**
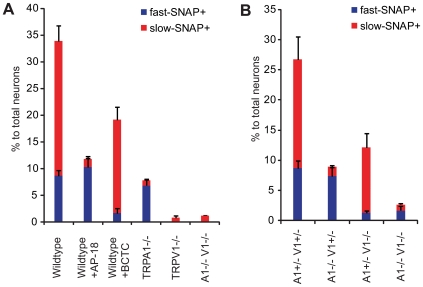
NO donor activates DRG neurons via TRPV1 and TRPA1. Proportion (%) of neurons activated by SNAP (3 mM) is shown and characterized as fast-SNAP+ (blue) and slow-SNAP+ (red) in different genetic backgrounds (e.g., A1^−/−^ V1^−/−^ represents TRPA1^−/−^ TRPV1^−/−^) and treatments. (A) DRG neurons were challenged with SNAP (3 mM) for 5 min, followed by capsaicin (Caps, 10 µM), mustard oil (MO, 0.5 mM), and/or ATP (100 µM) as controls (n≥4 each, see Table 1 for quantitation). AP-18 (100 µM) and BCTC (1 µM) were applied 3 min prior to SNAP application. (B) DRG neurons were first pretreated with forskolin (100 µM) and m-3M3FBS (50 µM) for 10 min, followed by the same protocol as (A). (n≥7 each, see Table 2 for quantitation). Haploinsufficiency has been reported for TRPA1 [58]–[59] when monitoring certain phenotypes and may influence the proportion of slow-SNAP+ neurons observed in our cultures.

Since either TRPV1 or TRPA1 could mediate NO donor-induced calcium influx into DRG neurons, we first tested whether specific antagonists for TRPA1 (AP-18: (Z)-4-(4-chlorophynyl)-3-methylbut-3-en-2-oxime, [35]) and TRPV1 (BCTC: N-(4-t-Butylphenyl)-4-(3-Chloropyridin-2-yl)tetrahydropyrazine-1(2H)-carboxamide, [36]) could inhibit DRG activation by SNAP. Interestingly, the slow-SNAP+ population (red bars) was specifically and dramatically reduced by the TRPA1 antagonist AP-18 (100 µM), while the fast-SNAP+ population was reduced by the TRPV1 antagonist BCTC (1 µM) (Fig. 4A and Table 1). AP-18 appeared to have little if any effect on the fast-SNAP+ population, while BCTC had little effect on the slow-SNAP+ population. These results suggest that TRPA1 and TRPV1 mediate slow-SNAP+ and fast-SNAP+ responses, respectively, demonstrating that native TRPV1 and TRPA1 are each required for normal NO sensing. The slow-onset and sustained intracellular calcium increase of NO-induced responses have been observed for TRPA1 in calcium imaging [27], [37]. Also note that the fast-SNAP+ population (∼10%) is significantly less than capsaicin-responsive population (usually 25–50% [38]), suggesting that SNAP only activates a subset of TRPV1-positive neurons (presumably, those expressing high TRPV1 levels). Most fast-SNAP+ cells (73.4±8.6%) displayed significant increases in intracellular calcium to capsaicin while significantly fewer (28.9±5.6%) slow-SNAP+ responded to capsaicin. Also, the average magnitude of the capsaicin responses were 2.0±0.1 fold (fast-SNAP+) and 1.3±0.1 fold (slow-SNAP+) (p<0.005), consistent with slow-SNAP+ responders having smaller capsaicin sensitivity after SNAP exposure (we did not observe any difference in the latency of SNAP-induced activation between slow-SNAP+ Caps+ and slow-SNAP+ Caps- cells [23]). A number of possible explanations could account for this difference including lower TRPV1 expression and/or enhanced tachyphylaxis of TRPV1 in this population. It should also be noted that TRPA1-mediated slow-SNAP+ neurons do not display fast activation, suggesting that TRPV1 functionality is not sufficient (presumably due to low TRPV1 levels) to be activated (at detectable levels) by SNAP in TRPA1-positive neurons, even though TRPA1 and TRPV1 are co-expressed [39].

To further confirm the results with specific antagonists, we performed calcium imaging experiments on primary DRG neurons from TRPV1- and/or TRPA1-deficient animals. As expected, the slow-SNAP+ population was specifically compromised in TRPA1^−/−^ DRG neurons (Fig. 4A and Table 1). Unexpectedly, however, both fast and slow SNAP+ populations were virtually absent in TRPV1^−/−^ DRG neurons (Fig. 4A and Table 1), in apparent contradiction to the pharmacological study using BCTC (Fig. 4A, Wildtype + BCTC), and to our observation that TRPA1 is responsive to NO donors in heterologous expression systems (Fig. 2, 3). These results could be explained if TRPA1 functionality in TRPV1^−/−^ DRG neurons is compromised due to lack of constitutive TRPV1 activity. Indeed, it has been recently reported that constitutive TRPV1 activity plays a role in stabilizing TRPA1 expression [40]. Not surprisingly, mice lacking both TRPV1 and TRPA1 also showed a virtual absence of NO responses (Fig. 4A).

As TRPA1 appears less functional in TRPV1^−/−^ DRG neurons, we reasoned that sensitizing TRPA1 might enable us to observe TRPA1-dependent NO responses in TRPV1^−/−^ DRG neurons. Since PKA and PLC signaling pathways are reported to sensitize TRPA1 responses in heterologous systems [41], we attempted to sensitize TRPA1 in TRPV1^−/−^ DRG neurons by incubating DRG neurons with the PKA activator forskolin and PLC activator m-3M3FBS (N-(3-Trifluoromethylphenyl)-2,4,6-trimethylbenzenesulfonamide; [42]) prior to SNAP application. Remarkably, after incubation with forskolin and m-3M3FBS, we were able to observe the slow-SNAP+ population in DRG neurons from TRPV1-deficient (TRPA1^+/−^ TRPV1^−/−^) animals (Fig. 4B and Table 2). The slow-SNAP+ population was absent but the fast-SNAP+ population was retained in TRPA1-deficient (TRPA1^−/−^ TRPV1^+/−^) animals, confirming that the fast-SNAP+ is mediated by TRPV1 (Fig. 4B and Table 2). Almost-complete reduction in SNAP+ neurons in TRPV1/A1-double deficient animals (TRPA1^−/−^ TRPV1^−/−^) suggest that these two ion channels account for calcium influx induced by SNAP, which is the main conclusion from these studies (Fig. 4).

**Table 2:** PKA and PLC pathways sensitize TRPA1 to NO donor.

%	SNAP	SNAP fast	SNAP slow	Capsaicin	Mustard Oil
**V1^+/−^ A1^+/−^**	26.7±3.6	8.7±1.2	18.0±3.8	20.1±2.9	
**V1^−/−^ A1^+/−^**	12.1±2.6	1.3±0.3	10.8±2.4	0.0±0.0	25.7±3.8
**V1^+/−^ A1^−/−^**	9.0±1.4	7.4±1.4	1.5±0.3	17.9±3.0	0.1±0.1
**V1^−/−^ A1^−/−^** *	2.7±0.8	1.7±0.7	1.0±0.2	0.0±0.0	0.2±0.1

Summary of the percent of responsive “sensitized” DRG neurons to SNAP (3 mM), capsaicin (10 µM), and mustard oil (0.5 mM) from mice of the indicated genotypes (V1 represents TRPV1 and A1 represents TRPA1). DRG neurons were incubated with forskolin (100 µM) and m-3M3FBS (50 µM) for 10 min prior to SNAP application.

* ATP (100 µM) was applied in the last of the experiment as a positive control. Approximately 20% of total DRG neurons were activated by application of ATP.

### Endogenous NO Activates DRG Neurons via TRPV1 and TRPA1

**Figure 5:**
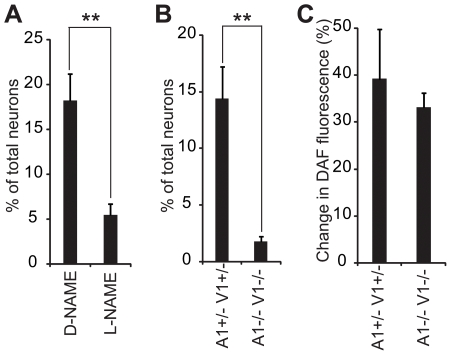
BH4 activates DRG neurons via TRPV1 and TRPA1. Proportion (%) of neurons activated by BH4 (1 mM) is shown. (A) DRG neurons pretreated with either L-NAME or D-NAME (200 µM, n = 7 each) were challenged with BH4 for 3 min. (B) DRG neurons from TRPA1^+/−^TRPV1^+/−^ (A1+/−V1+/−) or TRPA1^−/−^TRPV1^−/−^ (A1_−/−_V1_−/−_) animals were challenged with BH4 (1 mM) for 3 min. Capsaicin (10 µM) or ATP (100 µM) were applied at the end of the experiment as controls (n = 6 separate experiments, see Table 3 for quantitation). (C) Increases (%) in DAF fluorescence of DRG neurons of indicated genotypes are shown (n = 3 separate experiments comparing each genotype).

One criticism of the above experiments is that we are using high levels of NO donors, and that these levels are not physiologically relevant (also see Discussion). We therefore investigated whether TRPV1 and TRPA1 can be activated by endogenously-produced NO. BH4 (tetrahydrobiopterin), an essential cofactor for NO production by NOS, is known to increase intracellular calcium in DRG neurons at least partly in a Nitric Oxide Synthase (NOS)-dependent manner [19]. We observed that ∼15% of total DRG neurons displayed an increase in intracellular calcium upon BH4 (1 mM) application, and that L-NAME (*N*_ω_-Nitro-L-arginine methyl ester hydrochloride) but not its inactive isomer D-NAME (*N*_ω_-Nitro-D-arginine methyl ester hydrochloride) blocked the BH4-induced intracellular calcium increases (Fig. 5A and Table 3) as shown previously [19]. Importantly, the increase in intracellular calcium by BH4 is essentially absent in TRPV1^−/−^ TRPA1^−/−^ DRG neurons (Fig. 5B and Table 3), whereas BH4-induced production of NO levels were comparable between two genotypes as determined by the NO-sensitive fluorescence dye DAF-FM (4-amino-5-methylamino-2′,7′-difluorofluorescein) (Fig. 5C). The most straightforward conclusion from these studies showing the specific NOS inhibitor blocks BH4-mediated calcium increase is that TRPV1 and TRPA1 can be activated by endogenously-generated NO. However, it cannot be excluded that BH4's action on other pathways might play a role in increasing calcium levels.

**Table 3:** Endogenous NO activates DRG neurons via TRPV1 and TRPA1.

%	BH4	Capsaicin
**D-NAME**	18.2±2.9	38.1±3.0
**L-NAME**	5.5±1.2	40.6±2.8

%	BH4	Capsaicin	MO	ATP
**V1^+/−^ A1^+/−^**	14.4±2.8	25.6±1.8		
**V1^−/−^ A1^−/−^**	1.8±0.4	0.0±0.0	0.0±0.0	36.5±6.6

Summary of the percent of responsive DRG neurons to BH4 (1 mM), capsaicin (10 µM), mustard oil (0.5 mM), and ATP (100 µM) with indicated treatment (D-NAME or L-NAME, both at 200 µM), or from mice of indicated genotypes (V1 represents TRPV1 and A1 represents TRPA1).

### NO Donors Cause Pain via TRPV1 and TRPA1 *In Vivo*

**Figure 6:**
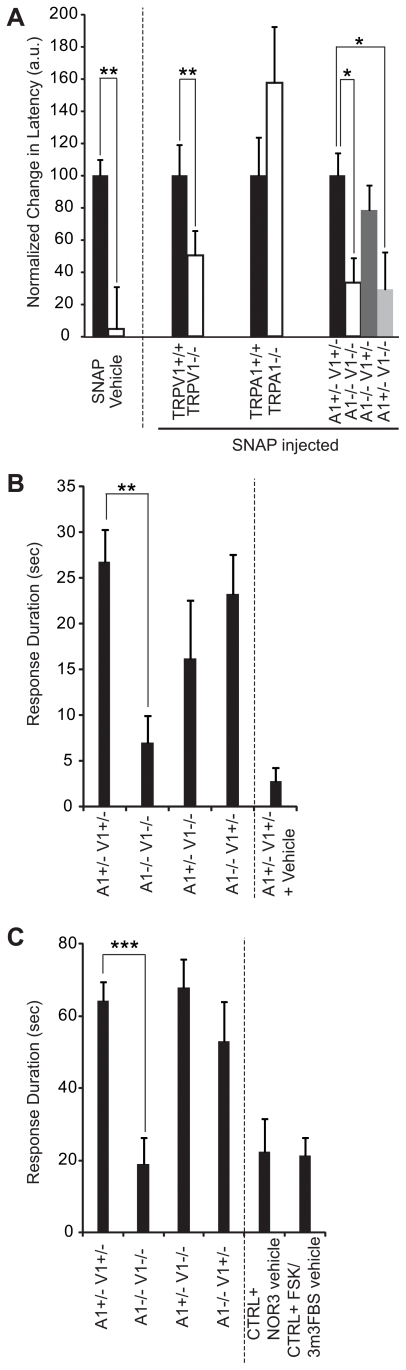
NO donors induce TRPV1-dependent thermal hyperalgesia and nocifensive behavior upon sensitization via TRPV1 and TRPA1. (A) Normalized changes in paw withdrawal latency are shown in response to a heat stimulus (average of three stimuli) 30 min after SNAP injection (25 mM in PBS, 1% DMSO). Decreases in paw withdrawal latency (%) were normalized to the value of SNAP-injected control mice (left columns) for each grouping (the larger the percentage, the faster the withdrawl to a heat stimulus). The first grouping represents SNAP- or vehicle- (1% DMSO) injected C57BL6 mice. The next three groupings show SNAP-induced thermal hyperalgesia in littermates with genotypes shown at bottom. Latencies of control animals were similar; 2.9±0.4 (SNAP-injected C57BL6), 2.7±0.3 (TRPV1^+/+^), 3.5±1.0 (TRPA1^+/+^), 3.4±0.6 (A1^+/−^ V1^+/−^). (B, C) Nocifensive response duration (flicking, licking, and lifting injected paw) recorded over 10 min after injection of NO donors in the hindpaw. N≥10 mice were tested (N≥6 for vehicle controls). Mice were first injected with forskolin and m-3M3FBS (1 mM each) followed by NOC7 (25 mM) (B) or NOR3 (1.5 mM) (C) injection. Genotype of animals is indicated (A1: TRPA1, V1: TRPV1). Vehicle-injected control mice ((B): A1^+/−^ V1^+/−^ + vehicle: vehicle injection 5 min after first injection, (C):CTRL+NOR3 vehicle: vehicle injection 15 min after first injection, and CTRL+FSK/3M3FBS vehicle: first injected with vehicle for forskolin and m-3M3FBS followed by NOR3 injection 15 min after first injection) are also shown. CTRL mice in (C) share the same genetic background and carry at least one copy of wildtype TRPV1 and TRPA1; the first four columns represent results from littermates.

We next investigated whether peripheral nociceptive actions of NO require TRPV1 and TRPA1. Intraplantar injection of NO donors (SNAP at 25 mM, NOR3 at 1.5 mM, NOC7: 3-(2-Hydroxy-1-methyl-2-nitrosohydrazino)-N-methyl-1-propanamine at 25 mM, or glyceryl trinitrate at 0.5 mg/ml) did not evoke significant acute pain behavior compared to vehicle responses [23]. However, intraplantar injection of SNAP (25 mM) induced thermal (but not mechanical) hyperalgesia in wildtype mice (Figure 6A, leftmost set of bars). TRPV1 is known to be required for induction of thermal hyperalgesia in response to a variety of stimuli. Indeed, SNAP-induced thermal hyperalgesia was largely TRPV1-dependent (Figure 6A).

Since acute pain was not observed after NO donor injection, we sought a protocol that would evoke NO donor-induced nociception after pharmacologically sensitizing nociceptors. Based on our DRG culture experiments, we tested whether pre-activation of PLC/PKA pathways could lead to NO donor-induced nocifensive behavior. After injection of forskolin and m-3M3FBS together (each at 1 mM), we observed significant nocifensive behavior upon injection of two chemically distinct NO donors (NOC7 (Fig. 6B) and NOR3 (Fig. 6C)) over background in wildtype animals. Remarkably, when we tested animals lacking TRPV1 and/or TRPA1, peripheral NO donor-induced pain behavior was compromised only when both TRPV1 and TRPA1 were ablated (Fig. 6B, C). This indicates that NO donor-induced nociception is dependent on both TRPV1 and TRPA1 (in another words, activation of either TRPV1 or TRPA1 by NO donors can cause acute pain after sensitization). We cannot say at this time if all NO-induced nociception is ablated in the double knockout mice, although the nocifensive response duration after vehicle injection in control animals is not statistically different from that after NO-donor injection in double knockout mice (Fig. 6B, C).

## Discussion

### Nitric Oxide Donors Activate TRPV1 and TRPA1

Our studies provide genetic and pharmacological evidence that NO donors activate TRPV1 and TRPA1 both in heterologous systems and in naïve DRG neurons. Previous calcium imaging studies on heterologously expressed TRPV1 and TRPA1 revealed NO donors evoked an increase in intracellular calcium levels dependent on channel expression [20]–[22]. It is presently not known whether the gating mechanism involves an NO-dependent shift in the voltage dependence of activation of TRPA1 and/or TRPV1, as demonstrated for TRPV1 and TRPM8 [43]–[44], and as shown for TRPA1 with regard to menthol dependent activation [29]. Regardless, NO appears to activate TRPV1 and TRPA1 through S-nitrosylation, which involves transfer of a nitric oxide group to cysteine sulfhydryls on proteins [5]. Consistent with this, we observe that C665, one of three TRPA1 cytoplasmic cysteines involved in responding to a variety of reactive chemicals [26], is also required for normal responsiveness to NO but not to the non-reactive agonist menthol implying that overall channel function of this mutation is not compromised. Although unlikely, we cannot rule out an indirect activation mechanism of TRPA1 via NO-induced formation of a membrane-delimited chemical or via other NO-activated redox-sensitive proteins (presumably present in detached patches of HEK293T, CHO, DRG cells). Furthermore, incomplete ablation of NO or iodoacetamide responses in single or triple TRPA1 cysteine mutations suggests that other mechanisms (residues) are also involved in sensing cysteine-reactive chemicals. Indeed, other amino acid residues (cysteines as well as histidines) of TRPA1 in addition to these three cysteines have recently been reported to be required for channel activation by zinc and intracellular base [31], [34], [45]. To date, how modification of cysteines in TRPA1 by reactive chemicals translates into channel activation is unknown. It is especially intriguing that activation appears to be independent of the shape of the chemical, or the type of the chemical reaction (alkylation, disulfide formation, Michael addition, nitrosylation, deprotonation etc.) [27]. It is possible that such reactions cause local or global conformational changes in the N-terminus cytoplasmic domain of TRPA1 that ultimately leads to channel gating.

There is increasing evidence that TRPV1 is also activated by reactive chemicals. However, while TRPA1 is promiscuous and is activated by most reactive chemicals, only a subset of such chemicals appears to activate TRPV1 [28], [32], [46]. We found that the cytoplasmic cysteine responsible for allicin-activation of TRPV1 is not required for NO-induced activation. Yoshida et al. proposed a general mechanism that two conserved cysteine residues among TRPs (including TRPV1) are required for activation by NO [20]. This finding has recently been challenged by Xu et al. who show that reduction of disulfide bonds between these two residues activates TRPC channels [33]. Therefore, whether NO targets these two pore cysteines in TRP channels is not settled. We show here that at least for TRPV1, the two pore cysteines are not required for activation by NO donor. It is possible that the large number of cysteine (18 Cys per subunit) and histidine (12 His per subunit) residues in TRPV1 cooperatively function in NO-sensing. More mechanistic studies are required to elucidate exactly how NO (and reactive chemicals in general) activates TRPV1, TRPA1, and other TRP channels; however, the focus of our study is on the genetic requirement of thermoTRPs in peripheral NO signaling in pain (below).

### TRPV1 and TRPA1 Mediate Nociception in Response to Peripheral Nitric Oxide

A role for peripherally-acting NO in hyperalgesia is supported by pharmacological data that NOS blockers relieve inflammatory and neuropathic pain in rodents [10]–[11]. Furthermore, NO donors cause acute moderate pain in humans, suggesting a role in acute nociception [14]. However, the behavioral consequences of direct peripheral application of NO donors have not been investigated in the mouse. Our studies reveal that NO donor injection in hindpaws causes TRPV1-dependent thermal hyperalgesia without causing significant acute pain behavior. These data suggest that direct comparison of human and rodent pain behavior is untenable and may point to a difficulty in assessing moderate pain in rodents. Note that due to solubility issues of NO donors, we were limited to using relatively low levels of NO donors for behavioral experiments (< ten fold the *in vitro* EC_50_). For capsaicin, injections of capsaicin at >1000 times the *in vitro* EC_50_ are typically used to observe acute pain behavior. Therefore, it is not surprising that significant acute nocifensive behavior was not observed, but only hyperalgesia. Regardless, we show that after PLC and PKA pathway activation (which sensitizes nociceptors, including TRPV1 and TRPA1 – see below), injections of two chemically distinct NO donors in the paw cause nociceptive behavior. Importantly, the NO donor-dependent nocifensive behavior required both TRPV1 and TRPA1, as we observed a decrease in nociception in mice lacking both TRPV1 and TRPA1, but not in individual knockout animals. This is consistent with our *in vitro* results showing that NO donors can activate both ion channels, and our DRG culture experiments, where we observe both TRPA1- and TRPV1-mediated responses. The most parsimonious explanation of these results is that NO's behavioral consequences is due to direct activation of these thermoTRPs; however, it cannot be ruled out that NO also targets TRPV1 and TRPA1 *in vivo* through indirect mechanisms. TRPV1 and TRPA1 are each well-characterized to be required for sensing a variety of noxious stimuli [47]; this is the first demonstration that mice lacking both genes have a shared requirement to respond to a specific stimulus, and therefore have overlapping functions. These two nocisensors have overlapping expression in a subset of DRG neurons [39] and thereby could potentially interact via intracellular signaling. Previous studies have shown that robust activation of TRPV1 can desensitize TRPA1 [48], but also that TRPV1 is required for proper TRPA1 expression [40]. These results point to elaborate communications between these two nocisensors.

### Potential Physiological Roles of NO-Induced TRPV1/TRPA1 Activation

Behavioral assays involving NO performed in this study are linked to peripheral sensitization: in one, NO donor causes thermal sensitization (Fig. 6A); in the other, sensitization reveals a nociceptive role for NO donors (Fig. 6B, C). Hence, the behavioral results highlight a physiological role of peripheral NO preferentially in the sensitized or hyperalgesic state. Indeed, NOS genes are known to be expressed in DRG neurons and are upregulated during inflammation and injury [8]–[9]. Moreover, macrophages, keratinocytes, and endothelial cells are known to release NO during inflammation, and are in close proximity to sensory nerve endings [2], [49]. Relevant to this, nitrooleic acid, an endogenous nitrated fatty acid which is generated during excessive NO production, was recently reported to covalently activate TRPA1 [50]. It is possible that NO and NO-derived compounds from many cell types act together on TRPV1 and TRPA1 during inflammation. Regardless, BH4-induced intracellular calcium increase in primary cultured DRG neurons is partly dependent on NOS [19], and essentially dependent on TRPV1 and TRPA1 (Fig. 5). It should be noted that we cannot exclude the possibility that BH4 acts on other pathways to regulate neuronal activity. Nevertheless, our results suggest that endogenous levels of NO are capable of activating thermoTRPs, and that TRPV1 and TRPA1 are involved in NO-dependent peripheral sensitization to pain. NO's action on TRPV1 and TRPA1 could potentially be relevant in other biological systems where NO and TRPV1/A1 have been implicated to be involved. This includes the trigeminovascular system (migraine) [51]–[52], synaptic plasticity in CNS [53]–[55], and relaxation in the bladder [56].

## Materials and Methods

### Ethics Statement

All experiments involving animals were conducted with the approval of The Scripps Research Institute Animal Research Committee. All aspects of the program for procurement, conditioning/quarantine, housing, management, veterinary care, and disposal of carcasses follow the guidelines set down in the NIH Guide for Care and Use of Laboratory Animals.

### Cell Culture/Ratiometric Calcium Imaging/FLIPR

#### Ratiometric calcium imaging

For determination of threshold for activation in ratiometric calcium imaging, the magnitude of the ratio change during exposure to agonist was normalized to the baseline ratio (fold-change) for each imaged neuron. A histogram of the fold change of each individual neuron in a particular experiment was constructed for each stimulus to determine the threshold for activation. The multimodal histogram contained one large peak around 1 (defined as the background response) and neurons with fold-changes greater than the first minimum (defined as threshold) were considered responsive. For DRG neurons, at least 200 neurons were selected in each experiment and for transiently transfected CHO cells, at least 30 YFP-positive cells were selected.

#### Primary DRG cultures

Extirpated thoracic and lumbar DRGs were dissociated by incubation for one hour at 37°C in a solution of culture medium (Ham's F12/DMEM with 10% Horse Serum, 1% penicillin-streptomycin) containing 0.125% collagenase (Worthington Biochemicals) followed by a 30 min incubation in 10 ml of culture media with 1.25 units papain. Cells were fully dissociated with a flame-polished pipette, and centrifuged (10 min, 1000 rpm, RT) with BSA column. Dissociated neurons were placed on poly-D-lysine/laminin Coated Glass Coverslips (BD Biosciences). Growth media was supplemented with 50 ng/ml nerve growth factor and 25 ng/ml glial cell line-derived neurotrophic factor.

#### Transient transfection

CHO or HEK293T cells were transiently transfected with plasmids encoding rat TRPV1 (rTRPV1), human (hTRPA1) or mouse (mTRPA1) TRPA1, and their mutants using FuGENE Transfection Reagent (Roche) according to manufacturer's instructions. CHO cells were used for ratiometric calcium imaging experiments since they are more adherent than HEK293T cells. HEK293T cells were used for FLIPR because of their high transfection efficiency and for electrophysiological experiments because they are more amenable to performing the different patch clamp configurations used in this study. For calcium imaging and electrophysiology, CHO or HEK293T cells were co-transfected with a pcDNA3-based YFP marker plasmid. Control cells were transfected with YFP vector alone. For FLIPR experiments, HEK293T cells were transiently transfected with plasmids using FuGENE Transfection Reagent without co-transfecting YFP marker plasmid.

#### FLIPR (fluorometric imaging plate reader)

For determination of concentration-response curves by FLIPR (Molecular Devices), HEK293T cells transiently transfected with plasmids encoding rTRPV1, hTRPA1, or their mutants were used. Two days after plating cells, cells were washed with assay buffer (1X Hanks' Balanced Salt Solution supplemented with 20 mM HEPES) and then loaded with Fluo-3 dye (Molecular Probes) according to manufacturer's instructions.

To enable comparison between clones at different expression levels, all responses were normalized to the peak response to menthol (for TRPA1) or capsaicin (for TRPV1) for the same clone within the same experiment. Concentration response curves were fit using Igor Pro (WaveMetrics Inc.) with a Hill equation model. All data points represent mean ± S.E.M.

### NO Imaging

DRG neurons were loaded with DAF-FM deacetate (Invitrogen, 10 µM) for 20 min in the imaging buffer solution (1X Hanks' balanced salt solution (HBSS), 10 mM HEPES). Images of DAF-FM loaded cells with the excitation wavelength at 488 nm were captured with a LCD camera using MetaFluor (Universal Imaging Corp.). For direct comparison of results from different experiments, the fluorescence intensity of individual cells in each experiment was normalized to the baseline. At least 200 DRG neurons were imaged per experiment and normalized fluorescence intensities of individual neurons were averaged.

### Electrophysiology

#### Cell-attached configuration of the patch clamp technique

Cells were maintained at 25 °C in a high extracellular K^+^ saline (“2 mM Ca^2+^/HighK^+^-ES”) containing (in mM): 136 KCl, 5 NaCl, 2 MgCl_2_, 2 CaCl_2_, 10 HEPES, pH 7.3 with NaOH. High extracellular K^+^ was used to shift the resting membrane potential toward 0 mV and avoid voltage dependent changes in TRP activity due to off target effects on endogenous background currents (the SNAP-induced conductances observed in whole cell experiments with HEK293T cells reversed at +15±5 mV, n = 3). The resting potential is likely to be close to zero since the reversal potentials of SNAP-induced TRPV1 and TRPA1 macroscopic or averaged currents were near zero in the cell-attach patch configuration when voltage ramp protocols were used. Pipettes were filled with either “0 mM Ca^2+^ ES” or “2 mM Ca^2+^ ES” (which contained (in mM): 136 NaCl, 5 KCl, 2 MgCl_2_, 2 CaCl_2_, 10 HEPES, pH 7.3 with NaOH) and had resistances of 3–15 Mohm when filled. Voltage ramp- or step-induced currents were acquired using the indicated protocols. Only patches containing relatively few channels were chosen for study in order to obtain blank sweeps during control and activated conditions. All data were acquired at 11.1 KHz and single channel records were filtered off-line at 3 KHz).

#### Excised inside-out configuration of the patch clamp technique

Voltage protocols were similar to those described for cell-attached patch experiments. Pipettes contained 2 mM Ca^2+^ ES. The bath was continuously perfused at **∼**26°C with a Cs-aspartate based intracellular saline which contained (in mM): 154 L-aspartate, 5 NaCl, 5 EGTA, 0.08 CaCl_2_, 10 HEPES, 2 methanesulfonate, pH to 7.3 with CsOH (50 nM free Ca^2+^).

#### Analysis of fold change in channel activity

Methods were similar to those described in [34]. To determine the fold change in control channel activity in the presence of agonist, blank sweeps (sweeps containing no TRP channel openings in the absence or presence of agonist) were used to determine the “leak”-subtracted control and agonist-induced level of activity at +120 mV (ramp protocol) or +100 mV (step protocol). The fold-change in activity was calculated by measuring the TRP-mediated current obtained by averaging consecutive traces acquired 1 to 2 min prior to agonist addition and during the maximal effect of agonist. Baseline currents were stable (<5% change in basal current) throughout the measurements.

#### Excised or cell-attached patch clamp data analysis

Data in all figures and text are shown as mean ± S.E.M. Statistical significance of the fold change in activity was evaluated using one sample *t*-test in combination with the Wilcoxon rank sum non-parametric test.

### Behavioral Assays

All experiments involving animals were conducted on littermate male mice 6–16 weeks old with the approval of The Scripps Research Institute Animal Research Committee. Animals were acclimated for an about one hour in the testing environment prior to experiments unless otherwise stated. Experimenters were blind to genotype.

#### Animals

All the genetically modified animals were tested and compared with their control (wildtype or double heterozygote) littermates. C57BL6/J mice were used only when comparing between wildtype animals. TRPV1^−/−^ mice were obtained from Jackson Laboratories (B6.129S4-*Trpv1^tm1Jul^*/J) backcrossed to C57BL6/J at least 10 generations. TRPA1^−/−^ mice were on mixed genetic background between C57BL6/J and 129/Ola (derived from E14 ES cell) and were kindly provided by Dr. David Corey (Harvard University).

#### Breeding strategy for TRPV1^−/−^ TRPA1^−/−^ double deficient animals

TRPV1 and TRPA1 double knockout animals were obtained as follows (V1:TRPV1, A1:TRPA1). First V1^−/−^ and A1^−/−^ animals were mated to generate V1^+/−^A1^+/−^ animals. V1^+/−^A1^+/−^ animals were then mated with each other to produce V1^+/−^A1^+/−^, V1^+/−^A1^−/−^, V1^−/−^A1^+/−^, and V1^−/−^A1^−/−^ animals. Finally, littermates from mating pairs of V1^+/−^A1^+/−^ with V1^−/−^A1^−/−^ or V1^+/−^A1^−/−^ with V1^−/−^A1^+/−^ were used for the behavioral assays.

#### Thermal hyperalgesia (hargreaves)

Thermal hyperalgesia assay was performed as described [57] using a Plantar IR Analgesia Meter (Ugo Basile 7370 Plantar Test). Baseline responses were measured one day prior to the administration of SNAP. Responses were measured post-injection in to the skin of left hindpaws with 10 µl of 25 mM SNAP or vehicle solution. Paw withdrawal latency to thermal stimuli (three stimuli per time point) were measured and averaged at 30 minutes post injection. 25 mM SNAP solution in PBS was prepared right before injection by dissolving 2.5 M stock in DMSO. Stock solution of SNAP was kept in dark.

#### Paw injection

NOC7: After acclimation, animals were first injected with 10 µl of forskolin and m-3M3FBS at 1 mM each (stock in ethanol). 5 min after the first injection, they were injected into the same spot with 10 µl of NOC7 at 25 mM (NOC7 was dissolved in 0.01 N NaOH, and then further dissolved into 1X PBS and 10 mM HEPES). Time spent on pain behavior (flicking, licking, and lifting injected paw) was recorded for 10 min after injection of NOC7.

NOR3: After acclimation, animals were first injected with 10 µl of forskolin and m-3M3FBS at 1 mM each (stock in DMSO). 15 min after the first injection, they were injected into the same spot with 10 µl of NOR3 at 1.5 mM (1% DMSO as vehicle). Time spent on pain behavior (flicking, licking, and lifting injected paw) was recorded for 10 min after injection of NO donor.

Initially, forskolin and m-3M3FBS (“sensitizers”) dissolved as stock solutions in DMSO were used for experiment using NOR3. Since DMSO injection causes acute pain behavior (solution of “sensitizers” contained 12% DMSO, which caused ∼60 sec pain behavior over 10 min after injection), we waited 15 min until cessation of the pain behavior. Subsequent experiments were performed using ethanol as a vehicle for the sensitizers when it was determined that ethanol produced no acute pain behavior. NOC7 was injected 5 min after sensitizer injection and behavior was determined.

### Site-Directed Mutagenesis

Point mutations were introduced using QuickChange Site-Directed Mutagenesis Kit (Stratagene) according to manufacturer's instructions.

### Chemicals

SNAP, Fura-2, and DAF-FM were purchased from Invitrogen. NOR3 and NOC7 were purchased from EMD Biosciences. BCTC and AP-18 were purchased from Biomol. All the other chemicals (capsaicin, mustard oil, forskolin, pCPT-cGMP, ruthenium red, ATP, NAP, potassium chloride, L-NAME, D-NAME, BH4, and m-3M3FBS) were purchased from Sigma-Aldrich. NO donors and BH4 were kept in the dark and prepared immediately before use.

### Data Analysis

Error bars represents S.E.M. for all figures and tables. P-values are based on one sample *t*-test in combination with the Wilcoxon rank sum non-parametric test for electrophysiological recordings (Fig. 3), on Student's *t*-test for comparing two samples (Fig. 5, 6A), and on one way ANOVA followed by Tukey's honest significance test for the remainder of experiments. * p<0.05, ** p<0.01, *** p<0.001

## References

[1] Stamler JS, Singel DJ, Loscalzo J (1992). Biochemistry of nitric oxide and its redox-activated forms.. Science.

[2] Lowenstein CJ, Dinerman JL, Snyder SH (1994). Nitric oxide: a physiologic messenger.. Ann Intern Med.

[3] Meller ST, Gebhart GF (1993). Nitric oxide (NO) and nociceptive processing in the spinal cord.. Pain.

[4] Koesling D, Russwurm M, Mergia E, Mullershausen F, Friebe A (2004). Nitric oxide-sensitive guanylyl cyclase: structure and regulation.. Neurochem Int.

[5] Hess DT, Matsumoto A, Kim SO, Marshall HE, Stamler JS (2005). Protein S-nitrosylation: purview and parameters.. Nat Rev Mol Cell Biol.

[6] Ahern GP, Klyachko VA, Jackson MB (2002). cGMP and S-nitrosylation: two routes for modulation of neuronal excitability by NO.. Trends Neurosci.

[7] Zochodne DW, Levy D (2005). Nitric oxide in damage, disease and repair of the peripheral nervous system.. Cell Mol Biol (Noisy-le-grand).

[8] Majewski M, Sienkiewicz W, Kaleczyc J, Mayer B, Czaja K (1995). The distribution and co-localization of immunoreactivity to nitric oxide synthase, vasoactive intestinal polypeptide and substance P within nerve fibres supplying bovine and porcine female genital organs.. Cell Tissue Res.

[9] Vizzard MA, Erdman SL, de Groat WC (1995). Increased expression of neuronal nitric oxide synthase in dorsal root ganglion neurons after systemic capsaicin administration.. Neuroscience.

[10] Boettger MK, Uceyler N, Zelenka M, Schmitt A, Reif A (2007). Differences in inflammatory pain in nNOS-, iNOS- and eNOS-deficient mice.. Eur J Pain.

[11] Guhring H, Gorig M, Ates M, Coste O, Zeilhofer HU (2000). Suppressed injury-induced rise in spinal prostaglandin E2 production and reduced early thermal hyperalgesia in iNOS-deficient mice.. J Neurosci.

[12] Meller ST, Dykstra C, Gebhart GF (1992). Production of endogenous nitric oxide and activation of soluble guanylate cyclase are required for N-methyl-D-aspartate-produced facilitation of the nociceptive tail-flick reflex.. Eur J Pharmacol.

[13] Kitto KF, Haley JE, Wilcox GL (1992). Involvement of nitric oxide in spinally mediated hyperalgesia in the mouse.. Neurosci Lett.

[14] Holthusen H, Arndt JO (1994). Nitric oxide evokes pain in humans on intracutaneous injection.. Neurosci Lett.

[15] Thomas DA, Ren K, Besse D, Ruda MA, Dubner R (1996). Application of nitric oxide synthase inhibitor, N omega-nitro-L-arginine methyl ester, on injured nerve attenuates neuropathy-induced thermal hyperalgesia in rats.. Neurosci Lett.

[16] Aley KO, McCarter G, Levine JD (1998). Nitric oxide signaling in pain and nociceptor sensitization in the rat.. J Neurosci.

[17] Ialenti A, Ianaro A, Moncada S, Di Rosa M (1992). Modulation of acute inflammation by endogenous nitric oxide.. Eur J Pharmacol.

[18] Hughes SR, Williams TJ, Brain SD (1990). Evidence that endogenous nitric oxide modulates oedema formation induced by substance P.. Eur J Pharmacol.

[19] Tegeder I, Costigan M, Griffin RS, Abele A, Belfer I (2006). GTP cyclohydrolase and tetrahydrobiopterin regulate pain sensitivity and persistence.. Nat Med.

[20] Yoshida T, Inoue R, Morii T, Takahashi N, Yamamoto S (2006). Nitric oxide activates TRP channels by cysteine S-nitrosylation.. Nat Chem Biol.

[21] Sawada Y, Hosokawa H, Matsumura K, Kobayashi S (2008). Activation of transient receptor potential ankyrin 1 by hydrogen peroxide.. Eur J Neurosci.

[22] Takahashi N, Mizuno Y, Kozai D, Yamamoto S, Kiyonaka S (2008). Molecular characterization of TRPA1 channel activation by cysteine-reactive inflammatory mediators.. Channels (Austin).

[23] Unpublished observations..

[24] Schmidtko A, Gao W, Konig P, Heine S, Motterlini R (2008). cGMP produced by NO-sensitive guanylyl cyclase essentially contributes to inflammatory and neuropathic pain by using targets different from cGMP-dependent protein kinase I.. J Neurosci.

[25] Wei JY, Cohen ED, Genieser HG, Barnstable CJ (1998). Substituted cGMP analogs can act as selective agonists of the rod photoreceptor cGMP-gated cation channel.. J Mol Neurosci.

[26] Hinman A, Chuang HH, Bautista DM, Julius D (2006). TRP channel activation by reversible covalent modification.. Proc Natl Acad Sci U S A.

[27] Macpherson LJ, Dubin AE, Evans MJ, Marr F, Schultz PG (2007). Noxious compounds activate TRPA1 ion channels through covalent modification of cysteines.. Nature.

[28] Salazar H, Llorente I, Jara-Oseguera A, Garcia-Villegas R, Munari M (2008). A single N-terminal cysteine in TRPV1 determines activation by pungent compounds from onion and garlic.. Nat Neurosci.

[29] Karashima Y, Damann N, Prenen J, Talavera K, Segal A (2007). Bimodal action of menthol on the transient receptor potential channel TRPA1.. J Neurosci.

[30] Ford PC, Lorkovic IM (2002). Mechanistic aspects of the reactions of nitric oxide with transition-metal complexes.. Chem Rev.

[31] Hu H, Bandell M, Petrus MJ, Zhu MX, Patapoutian A (2009). Zinc activates damage-sensing TRPA1 ion channels.. Nat Chem Biol.

[32] Bandell M, Macpherson LJ, Patapoutian A (2007). From chills to chilis: mechanisms for thermosensation and chemesthesis via thermoTRPs.. Curr Opin Neurobiol.

[33] Xu SZ, Sukumar P, Zeng F, Li J, Jairaman A (2008). TRPC channel activation by extracellular thioredoxin.. Nature.

[34] Dhaka A, Uzzell V, Dubin AE, Mathur J, Petrus M (2009). TRPV1 is activated by both acidic and basic pH.. J Neurosci.

[35] Petrus M, Peier AM, Bandell M, Hwang SW, Huynh T (2007). A role of TRPA1 in mechanical hyperalgesia is revealed by pharmacological inhibition.. Mol Pain.

[36] Valenzano KJ, Grant ER, Wu G, Hachicha M, Schmid L (2003). N-(4-tertiarybutylphenyl)-4-(3-chloropyridin-2-yl)tetrahydropyrazine -1(2H)-carbox-amide (BCTC), a novel, orally effective vanilloid receptor 1 antagonist with analgesic properties: I. in vitro characterization and pharmacokinetic properties.. J Pharmacol Exp Ther.

[37] Bandell M, Story GM, Hwang SW, Viswanath V, Eid SR (2004). Noxious cold ion channel TRPA1 is activated by pungent compounds and bradykinin.. Neuron.

[38] Caterina MJ, Leffler A, Malmberg AB, Martin WJ, Trafton J (2000). Impaired nociception and pain sensation in mice lacking the capsaicin receptor.. Science.

[39] Story GM, Peier AM, Reeve AJ, Eid SR, Mosbacher J (2003). ANKTM1, a TRP-like Channel Expressed in Nociceptive Neurons, Is Activated by Cold Temperatures.. Cell.

[40] Akopian AN, Ruparel NB, Jeske NA, Hargreaves KM (2007). TRPA1 Desensitization in Sensory Neurons is Agonist- Dependent and Regulated by TRPV1-Directed Internalization.. J Physiol.

[41] Wang S, Dai Y, Fukuoka T, Yamanaka H, Kobayashi K (2008). Phospholipase C and protein kinase A mediate bradykinin sensitization of TRPA1: a molecular mechanism of inflammatory pain.. Brain.

[42] Bae YS, Lee TG, Park JC, Hur JH, Kim Y (2003). Identification of a compound that directly stimulates phospholipase C activity.. Mol Pharmacol.

[43] Nilius B, Talavera K, Owsianik G, Prenen J, Droogmans G (2005). Gating of TRP channels: a voltage connection?. J Physiol.

[44] Malkia A, Madrid R, Meseguer V, de la Pena E, Valero M (2007). Bidirectional shifts of TRPM8 channel gating by temperature and chemical agents modulate the cold sensitivity of mammalian thermoreceptors.. J Physiol.

[45] Fujita F, Uchida K, Moriyama T, Shima A, Shibasaki K (2008). Intracellular alkalization causes pain sensation through activation of TRPA1 in mice.. J Clin Invest.

[46] Taylor-Clark TE, McAlexander MA, Nassenstein C, Sheardown SA, Wilson S (2008). Relative contributions of TRPA1 and TRPV1 channels in the activation of vagal bronchopulmonary C-fibres by the endogenous autacoid 4-oxononenal.. J Physiol.

[47] Patapoutian A, Tate S, Woolf CJ (2009). Transient receptor potential channels: targeting pain at the source.. Nat Rev Drug Discov.

[48] Jeske NA, Patwardhan AM, Gamper N, Price TJ, Akopian AN (2006). Cannabinoid WIN 55,212-2 regulates TRPV1 phosphorylation in sensory neurons.. J Biol Chem.

[49] Cals-Grierson MM, Ormerod AD (2004). Nitric oxide function in the skin.. Nitric Oxide.

[50] Taylor-Clark TE, Ghatta S, Bettner W, Undem BJ (2009). Nitrooleic acid, an Endogenous Product of Nitrative Stress, Activates Nociceptive Sensory Nerves via the Direct Activation of TRPA1.. Mol Pharmacol.

[51] Pietrobon D, Striessnig J (2003). Neurobiology of migraine.. Nat Rev Neurosci.

[52] Chizh Bea (2006). The TRPV1 antagonist SB705498 attenuates TRPV1 receptor-mediated activity and inhibits inflammatory hyperalgesia in humans: results from a Phase 1 study.. American Pain Society Meeting.

[53] Calabrese V, Mancuso C, Calvani M, Rizzarelli E, Butterfield DA (2007). Nitric oxide in the central nervous system: neuroprotection versus neurotoxicity.. Nat Rev Neurosci.

[54] Marsch R, Foeller E, Rammes G, Bunck M, Kossl M (2007). Reduced anxiety, conditioned fear, and hippocampal long-term potentiation in transient receptor potential vanilloid type 1 receptor-deficient mice.. J Neurosci.

[55] Gibson HE, Edwards JG, Page RS, Van Hook MJ, Kauer JA (2008). TRPV1 channels mediate long-term depression at synapses on hippocampal interneurons.. Neuron.

[56] Birder LA, de Groat WC (2007). Mechanisms of disease: involvement of the urothelium in bladder dysfunction.. Nat Clin Pract Urol.

[57] Moqrich A, Hwang SW, Earley TJ, Petrus MJ, Murray AN (2005). Impaired thermosensation in mice lacking TRPV3, a heat and camphor sensor in the skin.. Science.

[58] Kwan KY, Allchorne AJ, Vollrath MA, Christensen A, Zhang DS (2006). TRPA1 contributes to cold, mechanical, and chemical nociception but is not essential for hair-cell transduction.. Neuron.

[59] Bautista DM, Jordt SE, Nikai T, Tsuruda PR, Read AJ (2006). TRPA1 Mediates the Inflammatory Actions of Environmental Irritants and Proalgesic Agents.. Cell.

